# Supplementary material for: Incidence and risk factors of new-onset sacroiliac joint pain after spinal surgery: a systematic review and meta-analysis
Source: PeerJ. 2024 Sep 26;12:e18083. doi: 10.7717/peerj.18083 (PMC11439385; doi:10.7717/peerj.18083)
Supplement: Supplemental Information 3 [file peerj-12-18083-s003.zip › code/Code.docx]

**Code for（_ES _LCI _UCI）：**

gen noEvent_exp= sijptotal - events

gen noEvent_con= nosijptotal - _events

metan events noEvent_exp _events noEvent_con, or fixed second(random) effect(OR)

**Code for Incidence of new-onset SIJP(Fig 2)：**

gen p= NoofSIJP / total

gen se=sqrt(p*(1-p)/ total )

metan p se, lcols( proportionauthor year NoofSIJP total ) random second(fixed) forestplot(xlabel(0.1 0.2 0.3) dp(4) texts(140) astext(80) graphregion(margin(tiny)) aspect(0.5) title(Incidence of new-onset SIJP))

**Code for Subgroup analyses for incidence of new-onset SIJP(Fig 3)：**

format proportion se %10.4f

metan proportion se, lcols(subgroups studies patients ) rcols( se ) by( group ) effect(Proportion) nooverall nosubgroup forestplot(dp(4) texts(130) astext(80) xlabel(0 .1 .2 .3) graphregion(margin(tiny)) aspect(0.6) title(Subgroup analyses for incidence of new-onset SIJP))

**Code for Sex/female(Fig 4)：**

label variable _events "Events"

gen logor=log( _es )

gen loglci=log( _lci )

gen loguci=log( _uci )

gen selogor=((loguci-loglci)/(1.96*2))

metan logor loglci loguci, lcols( author year sijptotal events nosijptotal _events) fixed second(random) effect(OR) eform forestplot(texts(140) astext(80) graphregion(margin(tiny)) aspect(0.4) title(Sex/female))

sensitivity analysis:

metaninf logor selogor , label(namevar= author ,yearvar=year) fixed eform

**Code for Age(Fig 5)：**

label variable nn "N"

label variable _sd "SD"

format sijpmean sd nosijpmean _sd %10.2f

metan n sijpmean sd nn nosijpmean _sd , fixed second(random) nostandard lcols( author year n sijpmean sd nn nosijpmean _sd ) forestplot(texts(160) astext(80) graphregion(margin(tiny)) aspect(0.45) title(Age))

sensitivity analysis:

metaninf n sijpmean sd nn nosijpmean _sd ,label(namevar= author , yearvar=year) fixed

**Code for Preoperative diagnosis(Fig 6)：**

label variable _events "Events"

gen logor=log( _es )

gen loglci=log( _lci )

gen loguci=log( _uci )

gen selogor=((loguci-loglci)/(1.96*2))

metan9 logor loglci loguci, lcols( author year sijptotal events nosijptotal _events) fixed second(random) effect(OR) eform texts(175) astext(80) graphregion(margin(tiny)) aspect(1.2) nooverall by( subgroup) title(Preoperative diagnosis)

sensitivity analysis ( Sensitivity analysis for each subgroup was performed respectively) :

metaninf logor selogor , label(namevar= author ,yearvar=year) fixed/random eform

**Code for No. of surgical segments(Fig 7)：**

label variable _events "Events"

gen logor=log( _es )

gen loglci=log( _lci )

gen loguci=log( _uci )

gen selogor=((loguci-loglci)/(1.96*2))

metan logor loglci loguci, lcols( author year sijptotal events nosijptotal _events) random second(fixed) effect(OR) eform texts(160) astext(80) graphregion(margin(tiny)) aspect(0.6) nooverall by( subgroup) title(No. of surgical segments)

sensitivity analysis( Sensitivity analysis for each subgroup was performed respectively):

metaninf logor selogor , label(namevar= author ,yearvar=year) fixed/random eform

**Code for Fig 8：**

label variable _events "Events"

gen logor=log( _es )

gen loglci=log( _lci )

gen loguci=log( _uci )

gen selogor=((loguci-loglci)/(1.96*2))

metan logor loglci loguci, lcols( author year sijptotal events nosijptotal _events) fixed second(random) effect(OR) eform forestplot(texts(160) astext(80) graphregion(margin(tiny)) aspect(0.6) tit("Fusion to sacrum"))

sensitivity analysis:

metaninf logor selogor , label(namevar= author ,yearvar=year) fixed eform

**Code for Fig 9：**

label variable nn "N"

label variable _sd "SD"

format sijpmean sd nosijpmean _sd %10.2f

metan9 n sijpmean sd nn nosijpmean _sd , random second(fixed) nostandard lcols( author year n sijpmean sd nn nosijpmean _sd) texts(120) astext(80) graphregion(margin(tiny)) aspect(0.83) nooverall by( subgroup ) title(Preoperative spondylopelvic parameters)

label variable nn "N"

label variable _sd "SD"

format sijpmean sd nosijpmean _sd %10.2f

metan9 n sijpmean sd nn nosijpmean _sd , random second(fixed) nostandard lcols( author year n sijpmean sd nn nosijpmean _sd) texts(120) astext(80) graphregion(margin(tiny)) aspect(0.83) nooverall by( subgroup ) title(Postoperative spondylopelvic parameters)

sensitivity analysis( Sensitivity analysis for each subgroup was performed respectively):

metaninf n sijpmean sd nn nosijpmean _sd ,label(namevar= author , yearvar=year) fixed/random

**Code for Fig 10：**

gen logor=log( _es )

gen loglci=log( _lci )

gen loguci=log( _uci )

gen selogor=((loguci-loglci)/(1.96*2))

metafunnel logor selogor ,by( author ) aspect(0.54) graphregion(margin(tiny))

metabias6 logor selogor

**Code for Fig 11：**

label variable _sd "SD"

format premean sd postmean _sd %10.2f

metan9 n premean sd n postmean _sd , random second(fixed) nostandard lcols( n premean sd postmean _sd) texts(130) astext(50) graphregion(margin(tiny)) aspect(0.88) nooverall by( subgroup ) title(SIJP group) xlabel(-43.5,43.5)

label variable _sd "SD"

format premean sd postmean _sd %10.2f

metan9 n premean sd n postmean _sd , random second(fixed) nostandard lcols( n premean sd postmean _sd) texts(130) astext(50) graphregion(margin(tiny)) aspect(0.88) nooverall by( subgroup ) title(NoSIJP group) xlabel(-41.7,20.61)

sensitivity analysis( Sensitivity analysis for each subgroup was performed respectively):

metaninf n sijpmean sd nn nosijpmean _sd ,label(namevar= author , yearvar=year) fixed/random
