# Supplementary material for: Incidence and risk factors of new-onset sacroiliac joint pain after spinal surgery: a systematic review and meta-analysis
Source: PeerJ. 2024 Sep 26;12:e18083. doi: 10.7717/peerj.18083 (PMC11439385; doi:10.7717/peerj.18083)
Supplement: Supplemental Information 5 [file peerj-12-18083-s005.docx]

1.The rationale for conducting the systematic review / meta-analysis.

The rationale for conducting the systematic review and meta-analysis is the limited available studies and conflicted research findings from existing studies, so more evidence is necessary to identify the risk factors of new-onset SIJP after spinal surgery. And there are still no relevant meta-analyses and systematic reviews on new-onset SIJP after spinal surgery.

2.The contribution that it makes to knowledge in light of previously published related reports, including other meta-analyses and systematic reviews.

There is still only one systematic review of the incidence of SIJP after lumbosacral fusion that only performed single-arm meta-analysis without controlled meta-analysis and did not exclude relevant studies involving preoperative SIJP from the inclusion criteria.

This study conducted both qualitatively and quantitatively a comprehensive systematic review and meta-analysis of previous relevant studies to explore risk factors of new-onset SIJP after spinal surgery while providing evidence-based medical references for its early prevention, timely intervention, and appropriate treatment.
