# Supplementary material for: Incidence and risk factors of new-onset sacroiliac joint pain after spinal surgery: a systematic review and meta-analysis
Source: PeerJ. 2024 Sep 26;12:e18083. doi: 10.7717/peerj.18083 (PMC11439385; doi:10.7717/peerj.18083)
Supplement: Supplemental Information 6 [file peerj-12-18083-s006.docx]

Pubmed

#1 ("Sacroiliac Joint"[Mesh]) OR (Sacroiliac Joint[Title/Abstract])

#2 (low back pain[Title/Abstract] OR failed back surgery syndrome[Title/Abstract] OR back pain[Title/Abstract] OR flank pain[Title/Abstract] OR pelvic girdle pain[Title/Abstract] OR pain[Title/Abstract]) OR (low back pain[Mesh] OR failed back surgery syndrome[Mesh] OR back pain[Mesh] OR flank pain[Mesh] OR pelvic girdle pain[Mesh] OR pain[Mesh])

#3 (Diskectomy[Title/Abstract] OR spinal fusion[Title/Abstract] OR orthopedic procedures[Title/Abstract] OR fusion[Title/Abstract] OR decompression[Title/Abstract] OR spine surgery[Title/Abstract] OR lumbar surgery[Title/Abstract] OR decompress[Title/Abstract] OR decompressive[Title/Abstract] OR spinal surgery[Title/Abstract] OR postoperative[Title/Abstract] OR after surgery[Title/Abstract] OR surgery[Title/Abstract]) OR (Diskectomy[Mesh] OR spinal fusion[Mesh] OR orthopedic procedures[Mesh])

#4 #1 AND #2 AND #3

Cochrane total111，1review，81publication，39excluded by title

Web of science

((((((((((((((((((((((((((((((((((((((TI=(Diskectomy)) OR TS=(Diskectomy)) OR AB=(Diskectomy)) OR AB=(Spinal Fusion)) OR TS=(Spinal Fusion)) OR TI=(Spinal Fusion)) OR TS=(Orthopedic Procedures)) OR TI=(Orthopedic Procedures)) OR AB=(Orthopedic Procedures)) OR AB=(fusion)) OR TS=(fusion)) OR TI=(fusion)) OR TS=(Decompression)) OR TI=(Decompression)) OR AB=(Decompression)) OR AB=(spine surgery)) OR TS=(spine surgery)) OR TI=(spine surgery)) OR TS=(Lumbar surgery)) OR TI=(Lumbar surgery)) OR AB=(Lumbar surgery)) OR AB=(Decompress)) OR TI=(Decompress)) OR TS=(Decompress)) OR TS=(Decompressive)) OR TI=(Decompressive)) OR AB=(Decompressive)) OR AB=(spinal surgery)) OR TS=(spinal surgery)) OR TI=(spinal surgery)) OR TS=(Postoperative)) OR TI=(Postoperative)) OR AB=(Postoperative)) OR AB=(after surgery)) OR TS=(after surgery)) OR TI=(after surgery)) OR TI=(surgery)) OR TS=(surgery)) OR AB=(surgery)

((AB=(Sacroiliac Joint)) OR TS=(Sacroiliac Joint)) OR TI=(Sacroiliac Joint)

(((((TI=(Low Back Pain)) OR TI=(Failed Back Surgery Syndrome)) OR TI=(Back Pain)) OR TI=(Flank Pain)) OR TI=(Pelvic Girdle Pain)) OR TI=(Pain)

(((((TS=(Low Back Pain)) OR TS=(Failed Back Surgery Syndrome)) OR TS=(Back Pain)) OR TS=(Flank Pain)) OR TS=(Pelvic Girdle Pain)) OR AB=(Pain)

(((((AB=(Low Back Pain)) OR AB=(Failed Back Surgery Syndrome)) OR AB=(Back Pain)) OR AB=(Flank Pain)) OR AB=(Pelvic Girdle Pain)) OR AB=(Pain)

Embase

('low back pain':ti,ab,kw OR 'failed back surgery syndrome':ti,ab,kw OR 'back pain':ti,ab,kw OR 'flank pain':ti,ab,kw OR 'pelvic girdle pain':ti,ab,kw OR pain:ti,ab,kw) AND 'sacroiliac joint':ti,ab,kw AND (diskectomy:ti,ab,kw OR 'spinal fusion':ti,ab,kw OR 'orthopedic procedures':ti,ab,kw OR fusion:ti,ab,kw OR decompression:ti,ab,kw OR 'spine surgery':ti,ab,kw OR 'lumbar surgery':ti,ab,kw OR decompress:ti,ab,kw OR decompressive:ti,ab,kw OR 'spinal surgery':ti,ab,kw OR postoperative:ti,ab,kw OR 'after surgery':ti,ab,kw OR surgery:ti,ab,kw)
